# Administration of L-arginine plus L-citrulline or L-citrulline alone successfully retarded endothelial senescence

**DOI:** 10.1371/journal.pone.0192252

**Published:** 2018-02-07

**Authors:** Tomoe Tsuboi, Morihiko Maeda, Toshio Hayashi

**Affiliations:** 1 Department of Geriatrics, Nagoya University Graduate School of Medicine School of Health Sciences, Daiko-Minami, Higashi-ku, Nagoya, Aichi, Japan; 2 Chubu University Graduate School of Bioscience and Biotechnology, Matsumoto-cho, Kasugai, Aichi, Japan; University of Newcastle, UNITED KINGDOM

## Abstract

L-citrulline and L-arginine supplementation has been shown to have several beneficial effects on the cardiovascular system. Nitric oxide (NO) protects against the progression of atherosclerosis and is synthesized by nitric oxide synthase (NOS), which converts L-arginine (L-Arg) into L-citrulline (L-Cit). Our previous study revealed that chronic administration of a combination of L-Cit and L- Arg has a better therapeutic effect on high cholesterol-induced atherosclerosis in rabbits. We investigated how L-Arg and L-Cit affect endothelial function, aging and atherosclerosis. Following a 3-day stimulation of human umbilical venous endothelial cells (HUVECs) with high glucose (HG: 22 mM) and L-Arg (300 μM), L-Cit (300 μM) or L-Arg plus L-Cit (LALC: each 150 μM) supplementation, endothelial senescence and function were evaluated. These amino acids were also administered to dyslipidemic type 2 diabetic (ZDFM) rats fed a high cholesterol diet. They were fed L-Arg or L-Cit or LALC for four weeks. Aortic senescence was investigated by measuring senescence-associated ß-galactosidase (SA-ß-gal), telomerase activity, DNA damage and p16^INK4a^ protein expression. Only L-Cit and LALC supplementation retarded the HG-induced endothelial senescence, as evaluated by SA-ß-gal activity, a widely used marker of cellular senescence, p16^INK4a^ expression, a senescence-related protein, and DNA damage. Under HG conditions, L-Cit and LCLA restored telomerase activity to levels observed under normal glucose (NG) conditions. Under HG conditions, L-Cit decreased ROS production, as measured by CM-H_2_DCFDA and the expression of p67^phox^, a major component of NADPH oxidase. Under HG conditions, L-Cit and LALC increased NO production, as measured by DAF-2AM. Endothelial NO synthase (eNOS) and phosphorylated eNOS were decreased under HG conditions and L-Cit and LALC significantly increased these levels. Arginase 2 protein expression increased under the HG conditions, and L-Cit and LALC significantly attenuated this effect. In ZDFM rats, SA-ß-gal activity was detected on the aortic endothelial surface; however, L-Cit and LALC reduced these levels. L-Cit and LALC both decreased the proportion of senescent cells. Furthermore, treatment with LALC for 4 weeks increased plasma NO production. Therefore conclusively, L-citrulline supplementation rescued NO levels better than L-arginine supplementation by inhibiting ROS production and arginase 2 protein expression. Consequently, L-Cit and LCLA supplementation retaeded HG-induced endothelial senescence.

## Introduction

Aging is an important risk factor for cardiovascular diseases [1]. Cellular senescence limits the ability of cultured human cells to divide *in vitro* and is accompanied by phenotypic changes in gene expression, morphology, and function [2]. In aged animals, increased levels of proinflammatory molecules are expressed in senescent cells, suggesting that cellular senescence plays a role in the pathogenesis of atherosclerosis *in vivo* [3]. We previously observed and reported that endothelial senescence is important in the progression of atherosclerosis [4–6]. The shortening of telomeres, repetitious DNA sequences at the ends of eukaryotic chromosomes, is observed at the cellular level in various phases of the aging process. Telomere length represents the *biological* age of a cell, in contrast to its *chronological* age, and the shortening of telomere length suggests the onset of replicative senescence [7]. Cellular senescence is accompanied by an increase in senescence-associated ß-galactosidase (SA-ß-gal) (assayed at pH 6), and endogenous lysosomal ß-gal activity is a cellular senescence marker [8]. The free radical theory suggests that oxidative stress promotes senescence by causing telomere shortening through inactivation of the Src kinase family members [9]. Nitric oxide (NO) is a well-known signaling molecule that protects against the progression of atherosclerosis [10]. NO is synthesized by nitric oxide synthase (NOS), which converts L-arginine (L-Arg) into L-citrulline (L-Cit). L-Arg can also be synthesized from L-Cit in endothelial cells through the L-Cit/L-Arg recycling pathway [11]. The role of NO metabolism in senescence, especially in terms L-Arg and L-Cit is unknown.

Diabetes mellitus is a major cardiovascular risk factor, and diabetic atherosclerosis may progress as a result of an increase in reactive oxygen species (ROS) and a decrease in NO bioavailability due to a high plasma glucose level [12]. In elderly diabetic patients, the incidence of cardiovascular diseases is increased; however, the relationship between diabetes and endothelial senescence and function has not yet been elucidated [13–15].

The organic compound L-Cit is a water-soluble α-amino acid derived from citrullus, the Latin word for watermelon, and is a potent endogenous precursor of L-Arg and NO. L-Cit supplementation has beneficial effects on the cardiovascular system [16–18]. In rabbits, chronic supplementation of a combination of L-Cit and L-Arg has a better therapeutic effect on high cholesterol-induced atherosclerosis [17] and the short-term actions of a combination of L-Arg and L-Cit affect NO bioavailability and blood circulation [16]. However, the mechanism by which L-Arg, L-Cit and the combination of L-Arg and L-Cit supplementation affect endothelial function has not been investigated. Therefore, the purpose of this study was to evaluate the effect of L-Arg, L-Cit and a combination of L-Arg and L-Cit on endothelial function, aging and atherosclerosis. Furthermore, we examined the effect of L-Arg and L-Cit on high glucose (HG)-induced endothelial senescence.

## Materials and methods

### Materials

L-Arg and L-Cit were provided by Kyowa Hakko Bio Co., Ltd. (Tokyo, Japan). We purchased D-glucose and 4,5-diaminofluorescein diacetate (DAF2-AM) from Sigma-Aldrich (St. Louis, MO, USA), 5-dodecanoylaminofluorescein di-ß-D-galactopyranoside (C12FDG; 33 mM) from Molecular Probes (Eugene, OR, USA) and 5-(and-6)-chloromethyl-2ʹ, 7ʹ dichlorodihydrofluorescein diacetate, acetyl ester (CM-H2DCFDA; 10 μM) from Invitrogen (Carlsbad, CA, USA).

### Cell culture

Human umbilical venous endothelial cells (HUVECs) from Lonza Walkersville Inc. (Walkersville, MD, USA) were cultured in endothelial cell growth medium-2 prior to the experiments. During the experimental period, we cultured HUVECs in modified endothelial cell growth medium-2 that lacked insulin-like growth factor-1 (IGF-1) but contained 2% fetal bovine serum. We used sub-confluent, passage five to passage nine cells in the experiments, as in our previous study [5]. We harvested cells at sub-confluence and seeded them into six-well plates. Then, we stimulated the cells with 5.5 mM D-glucose (normal glucose; NG) or 22 mM D-glucose (high glucose; HG) for 3 days. L-Arg (300 μM), L-Cit (300 μM) or a combination of each amino acid at a half dose (150 μM) (LALC) was administered during the 3-day period of glucose stimulation.

To calculate a growth curve, HUVECs were stained with 4’6-diamidinophenylindole (DAPI; DAPI-Fluoromount-G (Southern-Biotech, USA)) for 10 min, and the total number of cells was counted under a KEYENCE BZ9000 immunofluorescence microscope (KEYENCE, Japan) before the start of amino acids stimulation (day 0) and after amino acids stimulation (day 1, day 2, day 3).

### Measurement of cellular senescence

SA-ß-gal activity was measured by flow cytometry as described previously [4–6]. After the experiment, the cells were incubated with C12FDG (33 mM) at 37°C for 2 hours. The cells were trypsinized and analyzed using a flow cytometer (FACS Calibur, Becton Dickinson, USA). Histochemical staining for SA-ß-gal was performed at pH 6.0 using a senescence detection kit (BioVision, CA, USA). The cells and rat vessels were fixed for 10 min in Fixative Solution and incubated overnight at 37°C without CO_2_ with fresh Staining Solution Mix.

### Quantification of DNA damage

DNA damage was quantified using the DNA Damage Quantification Kit-AP Site Counting- (#DK02, Dojindo, Japan). We used the QIAamp DNA Mini Kit (51304, QIAGEN, Germany) to purify the genomic DNA and resuspended the DNA at a concentration of 100 μg/ml. The DNA was labeled with an aldehyde reactive probe (ARP), and the number of apurinic/apyrimidinic (AP) sites in the DNA was determined as per the manufacturer’s instructions.

AP sites are one of the major types of DNA lesions formed during of the base excision and repair of oxidized, deaminated or alkylated bases. The level of AP sites in cells is a good indicator of DNA lesions and repair against chemical damage and cell senescence. This kit uses the ARP reagent, which reacts specifically with an aldehyde group, which is the open ring from of the AP sites. After treating the AP site-containing DNA with ARP reagent, the AP sites are tagged with biotin residues, which can be quantified using an avidin-biotin assay followed by colorimetric detection.

### Human telomerase activity assay

Telomerase activity was measured using the telomere repeat application protocol (TRAP) assay with the TeloTAGGG Telomerase PCR ELISA PLUS kit (Roche Diagnostics, Mannheim, Germany). This kit provides a way to perform a highly sensitive photometric enzyme immunoassay to detect “telomerase activity” [5]. We used 3 μg of total protein in the PCR reaction to measure telomerase activity and determined protein concentrations using an RC DC^TM^ protein assay kit (Bio-Rad, CA, USA).

Telomerase adds telomeric repeats (TTAGGG) to the 3’-end of the biotin-labeled synthetic P1-TS-primer. These elongation products, as well as the Internal Standard (IS) included in the same reaction vessel, are amplified by PCR using the primer P1-TS and the anchor-primer P2. The PCR products are split into two aliquots, denatured and hybridized separately to digoxigenin (DIG)-labeled detection probes specific for the telomeric repeats (P3-T) y. Immobilized amplicons are then detected with an antibody against horseradish peroxidase-conjugated digoxigenin (Anti-DIG-HRP) so that telomerase activity can be detected from PCR measurements.

### Analysis of ROS and superoxide generation

We examined ROS levels and superoxide generation, including hydrogen peroxide, the hydroxyl radical, and peroxynitrite, by flow cytometric analysis. We detected intracellular oxidant generation with the fluorescent probe CM-H_2_DCFDA [6]. We incubated cells with CM-H_2_DCFDA (10 μM) at 37°C for 30 min and then performed flow cytometry.

### Measurement of nitric oxide and its metabolites

We assessed intracellular NO with DAF-2DA, a diaminofluorescein-2 diacetate fluorescence probe (10 μM). Cells were incubated in the dark for 2 hours at 37°C with phenol red-free endothelial cell growth medium-2 containing 10 μM DAF-2 and were then washed twice with PBS, fixed with 2% paraformaldehyde for 3 minutes and washed twice again with ice-cold PBS. NO production analysis was carried out using an FSX-100 microscope (Olympus, Tokyo, Japan) with excitation and emission wavelengths of 460–495 nm and 510–550 nm, respectively. We recorded images using FSX-BSW software and measured the increases in the green fluorescence intensity of the distinct groups using Mac Biophotonic ImageJ software [7]. All images were taken during the first 3 seconds of light exposure to avoid fluorescence decay.

Nitric oxide metabolites (NO: NO^2-^ and NO^3-^) in the medium were measured using HPLC [15].

### Western blot analysis

Western blots were performed as previously described [6, 14]. The membrane was blotted with the indicated antibodies and was processed using chemiluminescence. We used the following commercially available primary antibodies: anti-human eNOS (#610297, BD Bioscience, USA) mouse monoclonal antibody (1:2,000), anti-human phosphorylated-eNOS (Ser-1177) (sc-12972, Santa Cruz, USA) rabbit polyclonal antibody (1:300), anti-human p22^phox^ (sc-20781, Santa Cruz, USA) rabbit polyclonal antibody (1:300), anti-human p16^INK4a^ (ab54210, Abcam, UK) mouse monoclonal antibody (1:200), anti-human arginase 2 (sc-20151, Santa Cruz, USA) rabbit polyclonal antibody (1:1000), anti-human p47^phox^ (sc-17845, Santa Cruz, USA) mouse monoclonal antibody (1:200), anti-human p67^phox^ (sc-384510, Santa Cruz, USA) mouse monoclonal antibody (1:200), and anti-human ß-actin (Abcam, Cambridge, UK) mouse monoclonal antibody (1:15,000). We used an anti-rabbit IgG, HRP-Linked antibody (#7074, Cell Signaling, USA) and an anti-mouse IgG, HRP-Linked antibody (#7076, Cell Signaling, USA) as secondary antibodies.

### Animal model

We purchased dyslipidemic type 2 diabetic rats (ZFDM-Hos: ZFDM-*Lepr fa*, *fa/fa and fa/+*) from Hoshino Laboratory Animals, Inc. (Ibaraki, Japan). They are derived from Zucker fatty rats and were generated by the repeated mating of male fatty (*fa/fa*) and female lean (*fa/+*) rats [19]. The rats develop T2DM and dyslipidemia after being fed a high fat diet (D12336; Research Diets, NJ, USA) for 2 weeks. New ZFDM rats, aged 7 weeks, were acclimatized for two weeks at 20±3°C with free access to water. We divided the rats into four groups (6 rats per group): the control group, the L-Arg group (2.0% L-Arg in drinking water), L-Cit group (2.0% L-Cit in drinking water), and LALC group (1.0% L-Arg and 1.0% L-Cit in drinking water). L-Arg, L-Cit, and the test agents were administered under the conditions of *ad libitum* feeding and the animals were provided free access to water for four weeks. We measured blood glucose levels and body weights once a week and the amount of food and water consumed three times a week. After four weeks the rats were sacrificed under isoflurane (2%) gas anesthesia, their blood and vessels were collected and the SA-ß-gal activity and NO metabolite (NOX: NO^2-^ and NO^3-^) levels were measured.

This study was carried out in strict accordance with the recommendations in the Guide for the Care and Use of Laboratory Animals of the National Institutes of Health. The protocol was approved by the Committee on the Ethics of Animal Experiments of Nagoya University. All surgery was performed under isoflurane gas anesthesia, and all efforts were made to minimize their suffering.

### Statistics

Data are presented as the mean ± standard deviation (SD). We performed statistical analyses using Statcel2 software. A one-way analysis of variance (ANOVA) followed by Tukey’s multiple comparison tests was performed where appropriate. Pairwise comparisons were performed using Student’s t-test. P values < 0.05 were considered statistically significant.

## Results

**Fig 1:**
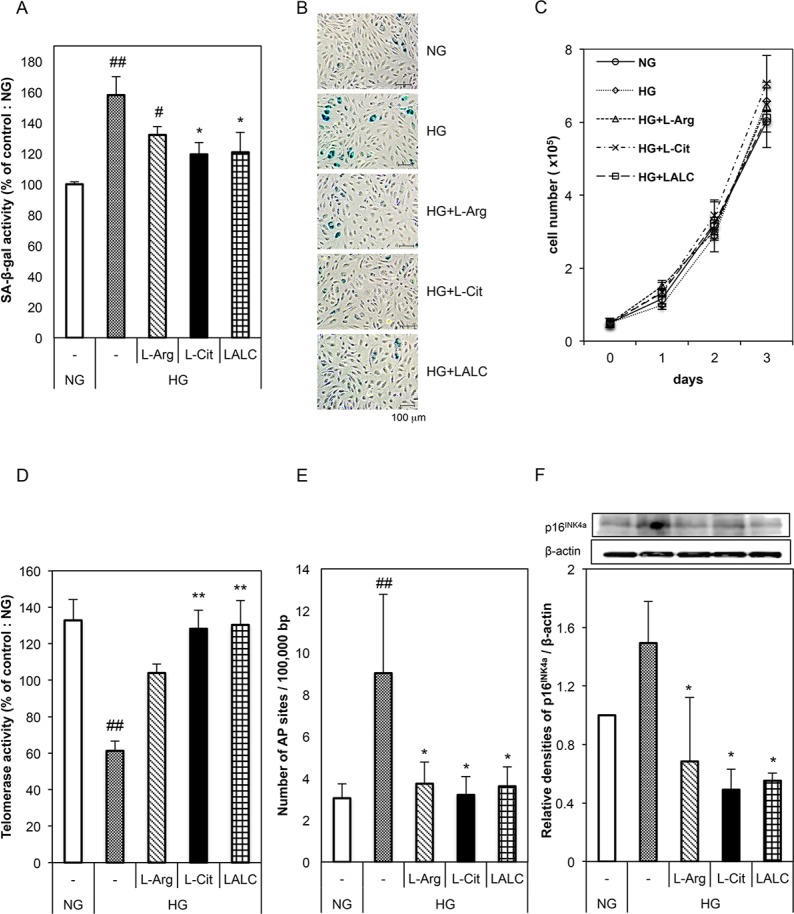
Effects of L-Arg, L-Cit and LALC on senescence in HUVECs. Cells were incubated with 5.5 mM glucose (NG) or 22 mM glucose (HG) for 3 days in the absence and presence of L-Arg (300 μM), L-Cit (300 μM) or LALC (150 μM each of L-Arg and L-Cit). (A) SA-ß-gal activity was measured to evaluate cellular senescence by flow cytometry (n = 5). (B) Representative photographs of SA-ß-gal staining (*blue;* SA-ß-gal positive cells). (C) Cellular growth curves determined by the counting dye method. All measures were performed in triplicate. (D) Telomerase activity was measured using the TRAP assay (n = 3). (E, F) The effect of L-Arg and/or L-Cit on DNA damage (as measured by the apurinic/apyrimidinic (AP) sites) (n = 4) (E) and a molecular marker of senescence (p16^INKa^ protein) (n = 3) (F). ##P<0.01, #P<0.05 versus NG. **P<0.01, *P<0.05 versus HG. Data are given as the mean ± SD from at least three independent experiments.

First, the effects of L-Arg and L-Cit on the senescence of HUVECs were investigated (Fig 1). We incubated cells for 3 days with 5.5 mM glucose (NG) or 22 mM glucose (HG). L-Arg (300 μM), L-Cit (300 μM) or LALC (150 μM each of L-Arg and L-Cit) was added to the HG medium. SA-ß-gal activity was significantly decreased in the L-Cit and LALC groups compared with the HG and L-Arg groups (Fig 1A and 1B). There was no difference in cell proliferation in the NG, HG and all groups of amino acid treatment (Fig 1C). We measured telomerase activity using the TRAP assay. Compared with the NG group, the HG condition significantly inhibited telomerase activity; however, L-Cit and LALC restored the telomerase activity level to that of the NG condition (Fig 1D). We also measured molecular senescence markers, DNA damage and p16^INK4a^ protein levels. DNA damage was quantified by determining the number of AP sites in the DNA (Fig 1E). Compared with the NG group, DNA damage in the HG group was significantly increased, whereas this effect was prevented by L-Cit and/or L-Arg treatment. Expression of the endothelial senescence-related protein, p16^INK4a^, was measured by western blot (Fig 1F). The HG condition increased the p16^INK4a^ level, while the NG and L-Cit and/or L-Arg conditions significantly decreased the p16^INK4a^ level compared with the HG condition. These results suggest that the HG condition induced endothelial senescence and that L-Cit and LALC reversed this senescence as detected by the decrease in molecular senescence markers, such as DNA damage and the p16^INK4a^ protein level. However, the growth curve data showed that the HG condition did not affect cell proliferation for at least 3 days.

**Fig 2:**
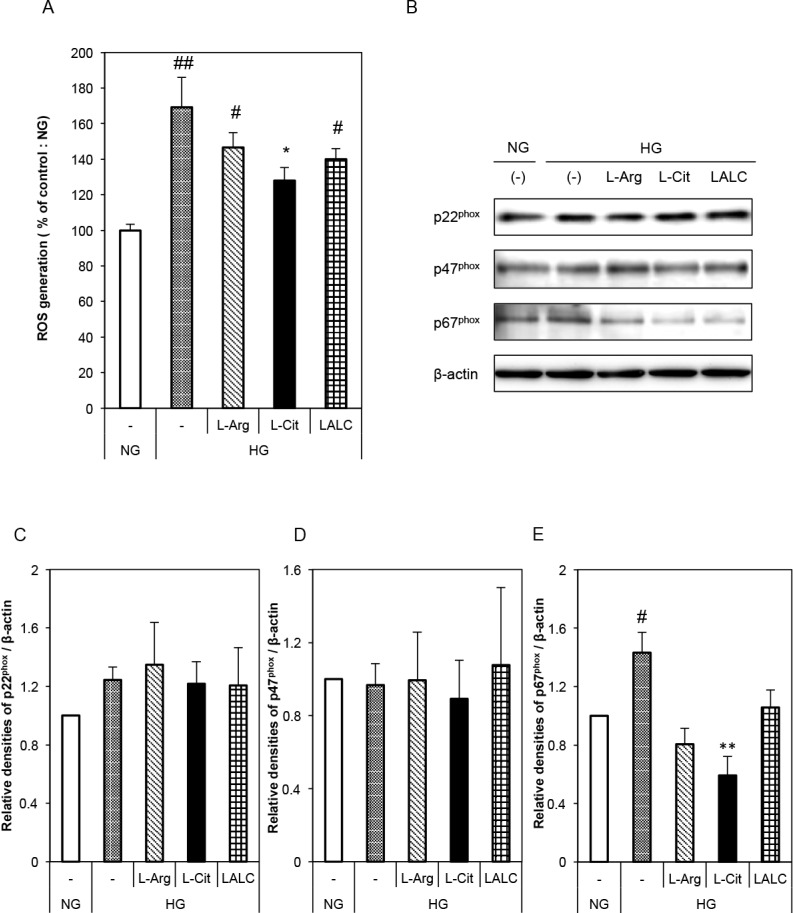
Effects of L-Arg, L-Cit and LALC on ROS generation in HUVECs. Cells were incubated with NG or HG for 3 days with or without L-Arg (300 μM) or L-Cit (300 μM). (A) ROS generation was detected as intracellular oxidant generation by flow cytometry (n = 6). (B) Typical western blots for p22^phox^, p47^phox^ and p67^phox^, a subunit of NADPH oxidase, are shown. (C-E) A summary of the quantification of densitometric measurements of the immunoblot data. The data were normalized to ß-actin (n = 3–5). ##P<0.01, #P<0.05 versus NG. **P<0.01, *P<0.05 versus HG. Data are given as the mean ± SD from at least three independent experiments.

The effects of L-Arg and/or L-Cit on ROS generation in HUVECs were investigated (Fig 2). We detected ROS generation by measuring intracellular oxidant generation by flow cytometry. HG significantly increased ROS generation and L-Cit significantly reduced the HG effect (Fig 2A). To investigate the source of the ROS, we measured the expression of subunits of NADPH oxidase, such as p22^phox^, p47^phox^ and p67^phox^, by Western blot (Fig 2B). NADPH oxidase is one of the most important superoxide sources in vascular cells. There was no difference in the expression levels of p22^phox^ and p47^phox^ (Fig 2C and 2D). Compared with the NG condition, the HG condition significantly increased the p67^phox^ protein level, while compared with the HG condition, the HG and L-Cit supplementation condition significantly decreased the p67^phox^ protein level (Fig 2E).

**Fig 3:**
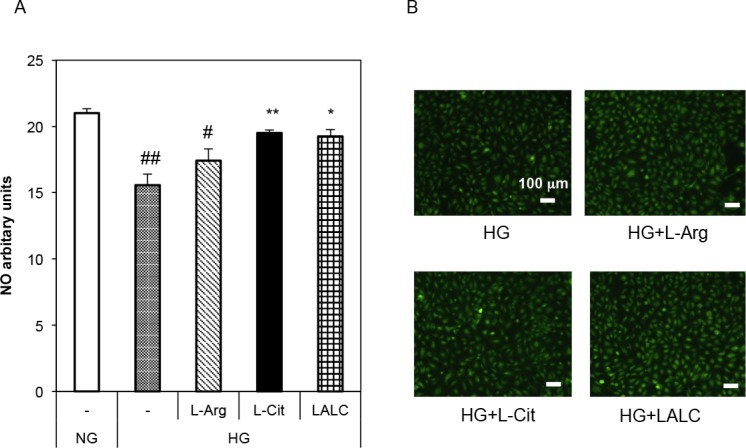
Effects of L-Arg, L-Cit and LALC on basal NO production in HUVECs. Cells were incubated with NG or HG for 3 days with or without L-Arg (300 μM) or L-Cit (300 μM). Intracellular NO production was assessed using DAF-2 DA, the NO-specific fluorescent dye, in HUVECs (n = 3). Fixed cell imaging of intracellular NO production in HUVECs and images were acquired for NO fluorescence. (A) The fluorescence intensity was measured. ##P<0.01, #P<0.05 versus NG. **P<0.01, *P<0.05 versus HG. Data are given as the mean ± SD of three independent experiments. (B) Fluorescent images of NO production in HUVECs (*green*; NO production).

The effects of L-Arg and L-Cit on basal NO production in HUVECs are shown in Fig 3. We assessed intracellular NO production in HUVECs using the NO-specific fluorescent dye DAF-2 DA under NG or HG conditions. Fixed cell imaging showed intracellular NO production in HUVECs (Fig 3A and 3B). The HG conditions significantly decreased NO production; however, L-Cit and LALC significantly reduced the effect of HG, and L-Arg partially reduced it.

**Fig 4:**
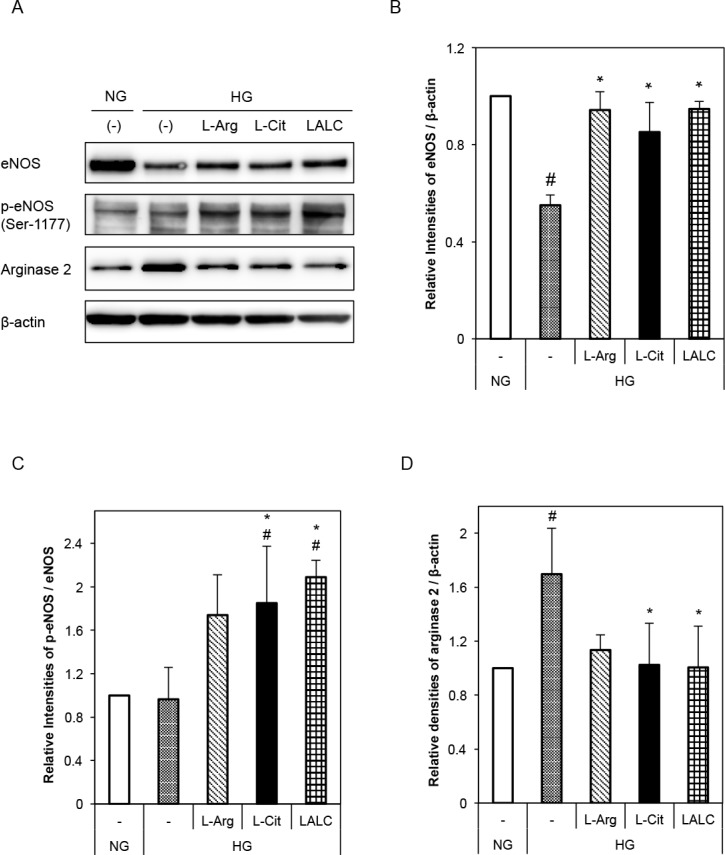
Effects of L-Arg, L-Cit and LALC on eNOS or arginase 2 expression in HUVECs. The cells were incubated with NG or HG for 3 days with or without L-Arg (300 μM) or L-Cit (300 μM). (A) Typical western blots for total eNOS, Ser-1177-phosphorylated eNOS and arginase 2 expression. (B-D) A summary of the densitometric measurements of the immunoblot data. The data were normalized to ß-actin (n = 3–5). #P<0.05 versus NG. *P<0.05 versus HG. Data are given as the mean ± SD of at least three independent experiments.

Next, we investigated the effects of L-Arg and/or L-Cit on NO generation in HUVECs. Fig 4 shows the effects of L-Arg and L-Cit on eNOS, phosphorylated eNOS (Ser1177) and arginase 2 expression in HUVECs under HG conditions (3 days of exposure). HG conditions decreased eNOS and tended to decrease phosphorylated eNOS protein levels (Fig 4B and 4C). However, L-Arg, L-Cit, and LALC almost completely decreased the effect of HG on eNOS and phosphorylated eNOS protein levels. Compared with the NG condition, the HG condition significantly increased arginase 2 protein levels, while the L-Cit and LALC conditions significantly decreased the influence of HG (Fig 4D).

**Fig 5:**
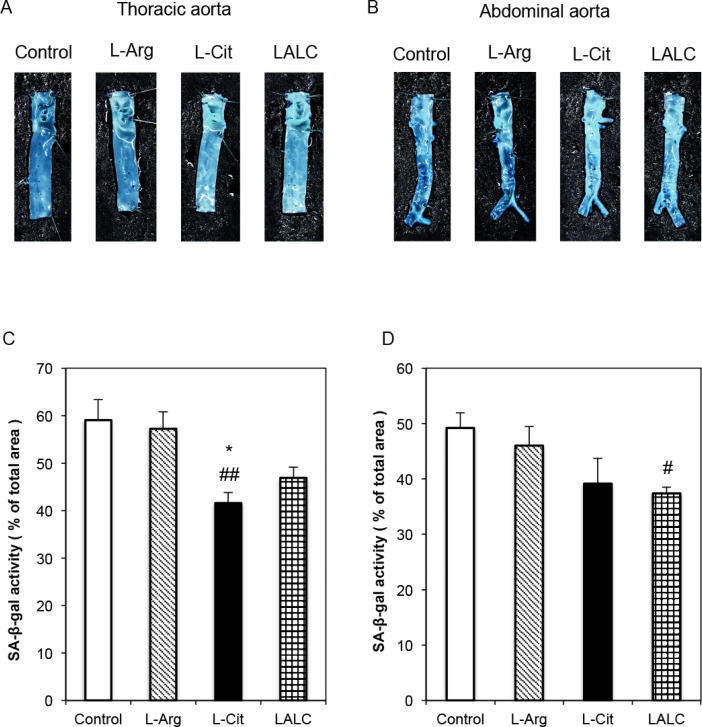
SA-ß-gal activity in the blood vessels of diabetic ZDFM rats. L-Arg, L-Cit and LALC supplementation for 4 weeks reduced SA-ß-gal staining. Representative photographs of SA-ß-gal-positive staining on the intimal side of the thoracic aorta (A) and abdominal aorta (B). The relative ratio of SA-ß-gal positively stained cells on the intimal side of the thoracic aorta (C) and abdominal aorta (D). Data are expressed as the mean ± SEM. (n = 6). *P<0.05 versus Arg. ##P<0.01, #P<0.05 versus Control.

Based on these *in vitro* effects of L-Arg and L-Cit, we performed animal experiments to investigate the effect of these amino acids on dyslipidemic type 2 diabetic rats (ZDFM rats). The rats were fed a high cholesterol diet without amino acid supplementation or with L-Arg, L-Cit or LALC for 4 weeks. Then, the internal surfaces (endothelial cells) of the thoracic and abdominal aorta were stained for SA-ß-gal activity (Fig 5). L-Arg and L-Cit treatment for 4 weeks reduced SA-ß-gal staining. Representative photographs of SA-ß-gal-positive staining in the endothelial cells of the thoracic aorta and abdominal aorta are shown in Fig 5A and 5B, respectively. In Fig 5C and 5D, the ratio of SA-ß-gal positively stained cells to SA-ß-gal negatively stained cells on the intimal side of the thoracic aorta and abdominal aorta, respectively, are shown. L-Cit and LALC decreased the proportion of senescent cells.

**Fig 6:**
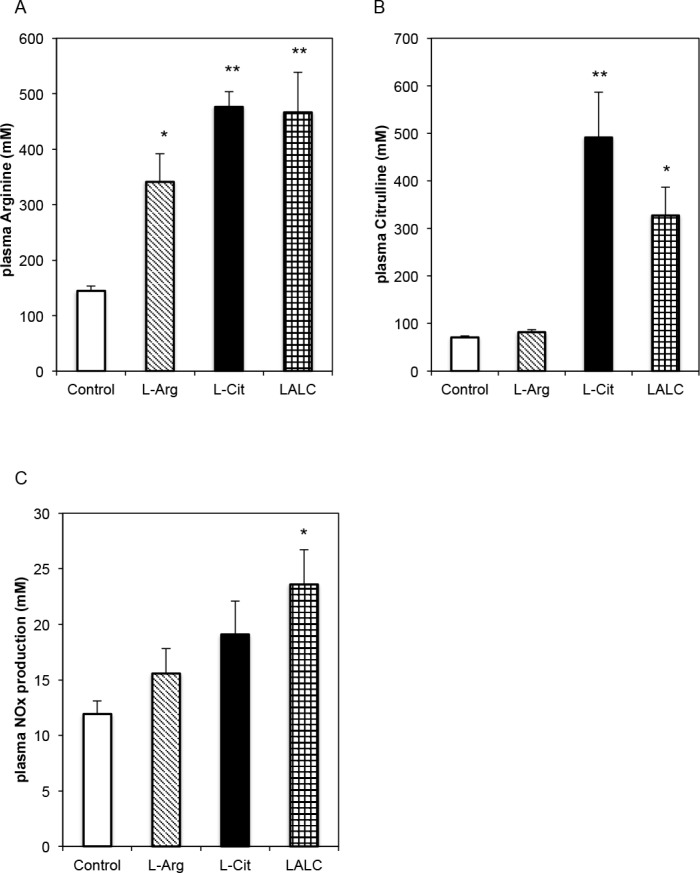
Plasma L-Arg, L-Cit and NO levels in diabetic ZDFM rats. L-Arg, L-Cit and LALC treatment for 4 weeks. (A) Plasma arginine. (B) Plasma citrulline. (C) Plasma NOx production. Data are expressed as the mean ± SEM. (n = 6). **P<0.01, *P<0.05 versus Control.

Fig 6 shows plasma L-Arg and L-Cit concentrations and the effects of L-Arg and L-Cit on diabetic ZDFM rat blood vessels in terms of endothelial function, such as the NO release capacity. Compared with the single amino acid supplementation, simultaneous administration of L-Cit and L-Arg increased plasma L-Arg levels; however, L-Arg supplementation did not increase the plasma L-Cit levels (Fig 6A and 6B). L-Arg and L-Cit supplementation for 4 weeks initially resulted in increased plasma NOx levels (Fig 6C).

## Discussion

We evaluated the effects of L-Arg and L-Cit on endothelial function, aging and atherosclerosis. L-Cit and LALC supplementation but not L-Arg supplementation reversed the endothelial senescence, DNA damage and p16^INK4a^; protein expression induced by HG conditions and restored telomerase activity, which was reduced by HG conditions in HUVECs. The levels of eNOS, phosphorylated eNOS, and NO metabolites were decreased under HG conditions; however, L-Cit and LALC restored the levels of these molecules. Dyslipidemic and diabetic rats (ZDFM rats) were fed a high cholesterol diet without amino acid supplementation or with L-Arg, L-Cit or LALC supplementation for four weeks. The aortic endothelial cells showed SA-ß-gal activity under HG conditions; however, treatment with L-Cit or LALC reduced this activity. The objective of this animal experiment was to identify the effect of long-term ingestion of oral L-Cit plus L-Arg on endothelial aging and the involvement of the NO-cGMP pathway in endothelial aging in a diabetic rat model.

The present study showed that NO metabolites and p-eNOS were decreased under the HG condition and L-Cit and LALC restored the levels of these molecules. NO is synthesized from L-Arg by eNOS via the L-Cit/L-Arg recycling pathway [16, 17, 18]. Although L-Arg supplementation has been reported to improve endothelial dysfunction in humans and animals [16, 20, 21], recent reports show no effects of chronic L-Arg supplementation on endothelial function because most orally administered. L-Arg is trapped in hepatic tissue following absorption by the gastrointestinal tract and is then catabolized by arginase 1 and 2 [22, 23, 24]. In contrast, L-Cit has been shown to be an effective precursor of L-Arg that serves as a sustained source of L-Arg for eNOS activity in human arteries [17, 18, 24]. L-Cit is an allosteric inhibitor of arginase activity [25]. L-Cit can pass through the gastrointestinal tract and arrive at the liver without being affected by intestinal or hepatic first-pass effects, likely due to the inhibition of arginase activity by L-Cit. Long term L-Arg supplementation up-regulated the arginase 2 protein expression and accelerated cellular senescence [26]. L-Cit and LALC supplementation significantly decreased arginase 2 protein levels. These results suggest that endothelial L-Cit inhibited arginase 2 protein expression and induced eNOS activity and up-regulated NO generation to restore endothelial senescence. Our data suggest that not only L-Arg but also L-Cit is important to produce NO and, L-Arg bioavailability.

Intracellular endothelial L-Arg is unavailable for NO production as cytosolic L-Arg availability for eNOS is limited by its sequestration in the plasmalemmal caveolae, where the L-Cit to L-Arg recycling pathway, which utilizes eNOS, is located [16, 25]. Therefore, the LALC combination markedly increased the plasma L-Arg level, thus increasing NO production. L-Cit is transported into endothelial cells by the neutral amino acid system-N transporter 1 (SN1) [27]. L-Cit may be directly taken up by endothelial cells through SN1, where it serves as a substrate for the production of L-Arg via the L-Cit/L-Arg recycling pathway and up-regulates eNOS activity. Long-term LALC supplementation significantly restored impaired endothelial-dependent vasodilation and dramatically reversed atherosclerotic lesions in rabbits [20]. LALC supplementation increased eNOS protein expression and thus represents one possible mechanism for the described effects [20]. Many studies have reported that endothelial dysfunction, which is strongly associated with decreased NO bioavailability, is a factor in the development of cardiovascular diseases [28].

Our data support the important clinical effects of L-Cit supplementation on vascular function. The free radical theory of aging posits that replicative senescence is predominantly due to the cumulative effect of ROS [4, 9, 29]. Increased NOX-derived ROS generation is involved in the development of endothelial dysfunction in many different conditions [30] and has been implicated in experimental heart failure. The active NOX2 oxidase complex contains not only NOX2 and its partner subunit p22^phox^ but also several regulatory subunits (p47^phox^, p67^phox^, Rac), whose association with the oxidase is required for its activation; p67^phox^ is essential for oxidase activation [31]. The endothelial expression of p67^phox^, a critical component of the superoxide-generating NADH/NADPH oxidase system, was significantly increased in the HG condition and decreased by L-Cit supplementation. We previously showed that the HG condition did not alter the expression level of p22^phox^, but intermittent HG increased ROS generation via increased expression of p22^phox^ [15]. Our data suggest that the ROS generated by the HG condition is caused by NOX2 via increased expression of p67^phox^. Our data also suggest that L-Cit decreases the influence of HG by decreasing p67^phox^ protein expression.

We observed an association between increased oxidative stress and decreased telomerase activity in the present study. Individuals with shorter telomere lengths in their white blood cells have been shown to have a 2.8-fold higher coronary risk than individuals in the highest quartile of telomere length in their white blood cells after adjusting for age [32]. Furthermore, the introduction of human telomerase reverse transcriptase (hTERT) into human cells extended both their lifespan and their telomere lengths to those typical of young cells [4, 33]. Both transcriptional and posttranslational mechanisms are involved in the regulation of hTERT. Transcriptional regulation functions as a regulatory mechanism in cancer cells [34] and kinases in endothelial cells, such as protein kinase C (PKC), ERK1/2 and Akt, regulate telomerase activity post-transcriptionally [35]. ROS activates kinases in the Src family and reduces the expression of Akt, thus inducing aging in endothelial cells. Further, phosphorylation of nuclear hTERT by Akt maintains its active status; however, the activation of Src family kinases induces the nuclear export of hTERT, decreasing the ability of hTERT to maintain telomere length and regulate aging [4]. We found that enhancement of ROS formation decreased telomerase activity prior to the onset of replicative senescence.

Atherosclerosis is an inflammatory disease that is typically preceded by endothelial dysfunction, oxidative stress [36] and a decline in NO production [1, 37]. Diabetic macroangiopathies, such as coronary atherosclerosis, also occur under nearly conditions, with an increase in superoxide anion production and a decrease in NO released by endothelial cells [28, 32]. Accordingly, our data suggest that the administration of LALC or L-Cit alone successfully retards endothelial senescence and reduces endothelial dysfunction induced by HG conditions.

We investigated the effect of L-Arg and L-Cit on endothelial function, aging and atherosclerosis, and our study demonstrated that L-Cit and LALC improved endothelial senescence. L-Cit and L-Arg scavenge ROS, reverse endothelial senescence and reduce the increase in ROS that is a coronary risk factor. L-Cit rescued NO levels better than L-Arg by the inhibition of ROS and arginase 2 protein expression and L-Cit and LALC supplementation reversed HG-induced endothelial senescence.

## Supporting information

S1 Fig **The changes in body weight (A) and blood glucose levels (B) in diabetic ZDFM rats. **ZDFM rats were administered L-Arg (■), L-Cit (△), or a combination of each at half dosage (LALC) (◇) by oral gavage.
(PPTX)
