# Supplementary material for: Administration of L-arginine plus L-citrulline or L-citrulline alone successfully retarded endothelial senescence
Source: PLoS One. 2018 Feb 7;13(2):e0192252. doi: 10.1371/journal.pone.0192252 (PMC5802914; doi:10.1371/journal.pone.0192252)
Supplement: S1 Fig — The changes in body weight (A) and blood glucose levels (B) in diabetic ZDFM rats. ZDFM rats were administered L-Arg (■), L-Cit (△), or a combination of each at half dosage (LALC) (◇) by oral gavage. (PPTX) [file pone.0192252.s001.pptx]

## Slide 1
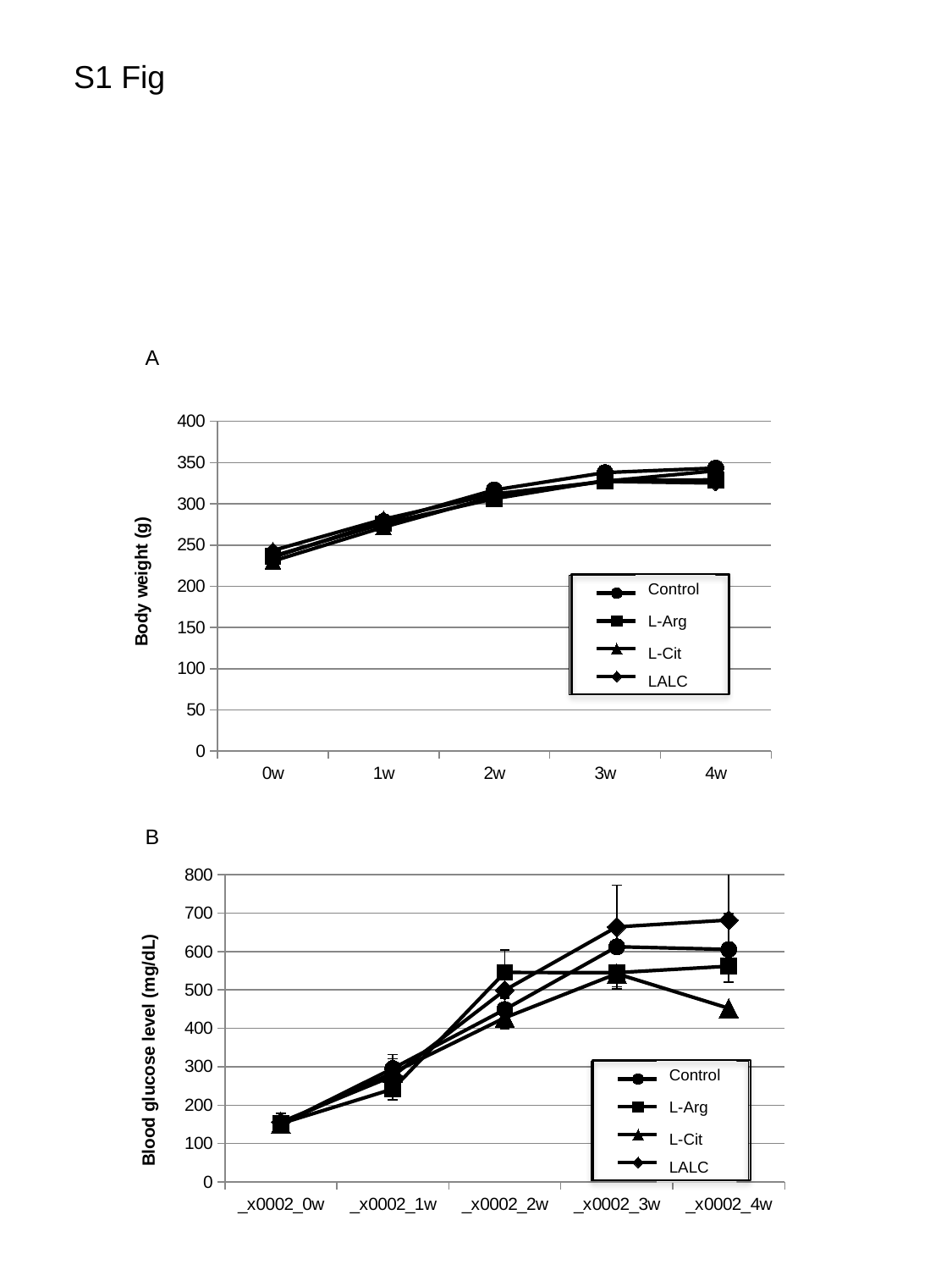

S1 Fig
A
### Chart
| Category | Cont. | Arg | Cit. | Arg+Cit |
|---|---|---|---|---|
| 0w | 235.1666666666667 | 236.5 | 230.5 | 243.3333333333333 |
| 1w | 278.5 | 275.8333333333333 | 271.6666666666666 | 281.3333333333333 |
| 2w | 316.8333333333333 | 306.1666666666666 | 308.8333333333333 | 311.8333333333333 |
| 3w | 337.8333333333333 | 328.1666666666666 | 327.3333333333333 | 327.1666666666666 |
| 4w | 343.3333333333333 | 329.0 | 340.0 | 325.1666666666666 |Control
L-Arg
L-Cit
LALC
B
### Chart
| Category | Cont. | Arg | Cit | Arg+Cit |
|---|---|---|---|---|
| _x0002_0w | 150.5 | 152.1666666666667 | 151.3333333333333 | 155.8333333333333 |
| _x0002_1w | 295.5 | 242.0 | 284.5 | 275.8333333333333 |
| _x0002_2w | 449.1666666666666 | 545.6666666666666 | 427.5 | 499.3333333333333 |
| _x0002_3w | 612.5 | 545.0 | 542.3333333333333 | 664.1666666666666 |
| _x0002_4w | 605.5 | 561.8333333333333 | 452.1666666666666 | 682.0 |Control
L-Arg
L-Cit
LALC
